# Supplementary figures and images for: How immunity shapes the long-term dynamics of influenza H3N2
Source: PLoS Comput Biol. 2025 Mar 20;21(3):e1012893. doi: 10.1371/journal.pcbi.1012893 (PMC11964465; doi:10.1371/journal.pcbi.1012893)

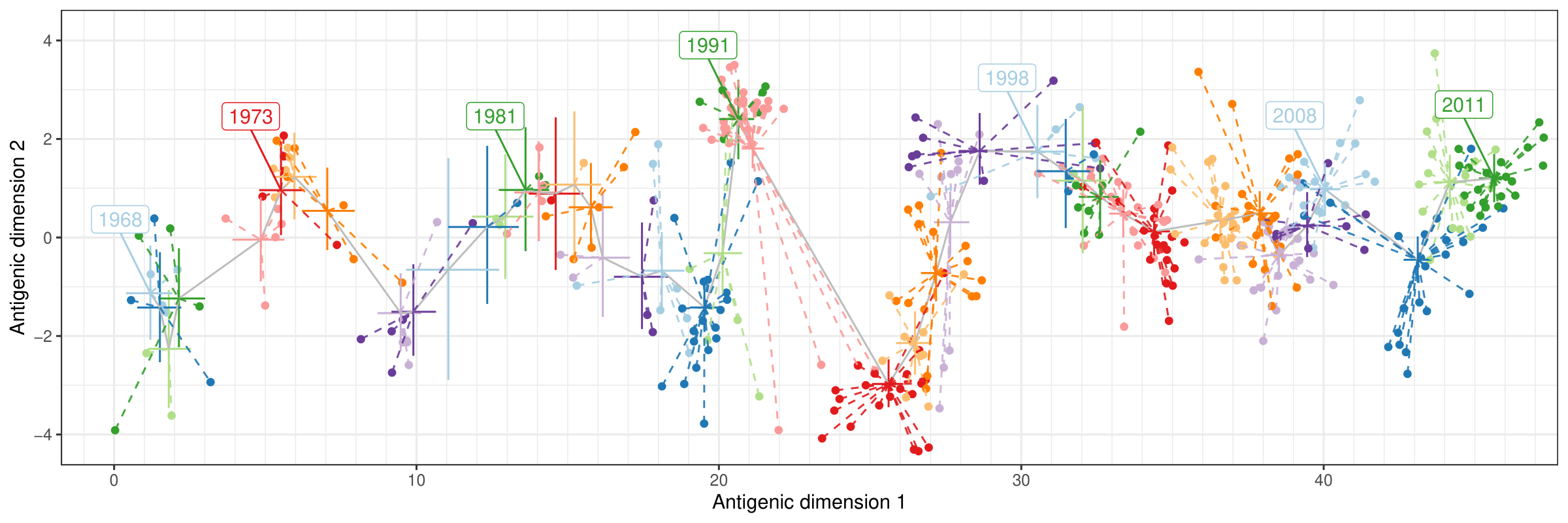

Supplement: S1 Fig — The antigenic distance between two strains (points) quantifies their dissimilarity, with one unit of antigenic distance corresponding to a two-fold dilution in hemagglutinin inhibition titre. Strains (points) are coloured by the year in which they were sequenced, and some years have been explicitly labelled for clarity. The statistical model describing global antigenic drift (see Methods) has been fitted to this antigenic map. The posterior of the mean antigenic coordinates, n = (n,x, n,y), for each year are shown with the posterior mean (centre of cross) and 95% confidence interval in antigenic dimension 1 (horizontal line of cross) and antigenic dimension 2 (vertical line of cross). Grey lines connect the mean antigenic coordinates between adjacent years; note that the mean antigenic coordinates increase along antigenic dimension 1 each year (from left to right). The strains associated with a given year are connected to the mean antigenic coordinates for that year by dashed lines, and are coloured the same. Note that in some years there are no strains on the antigenic map (e.g., 1978 [the second light blue year]). (TIFF) [file pcbi.1012893.s001.tiff]

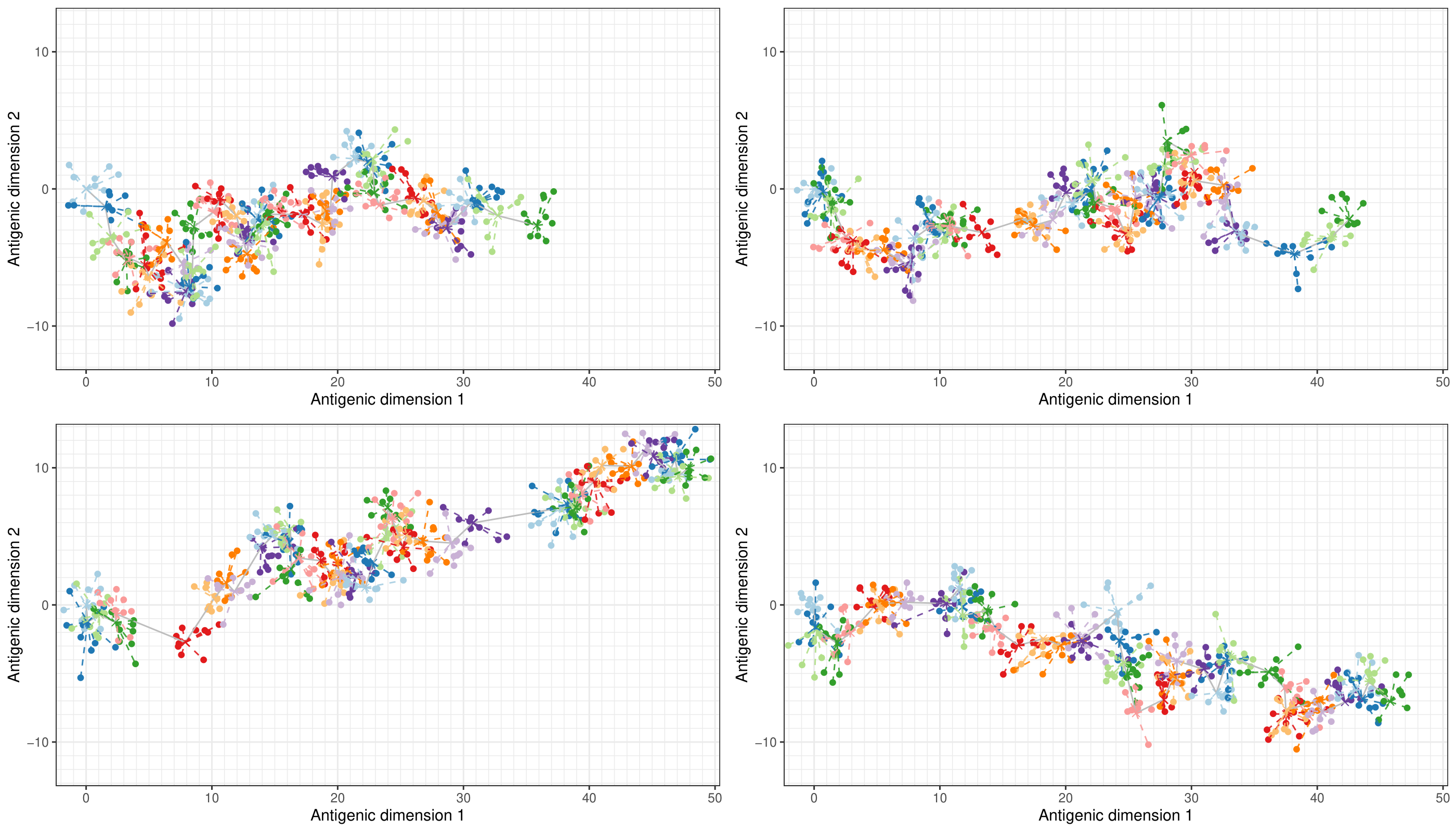

Supplement: S2 Fig — The simulated antigenic diversity of influenza strains for 44 years (colours highlight separate years with 10 strains per year). Strains are located on a two-dimensional antigenic map. The antigenic distance between two strains quantifies their dissimilarity, with one unit of antigenic distance corresponding to a two-fold dilution in hemagglutinin inhibition titre. Strains in a single year (points) are normally distributed around mean antigenic coordinates (dotted lines connect strains in a given year to the mean antigenic coordinates). The mean antigenic coordinates change each year (grey line connecting adjacent years), always progressing along antigenic dimension 1 (left to right). The mean antigenic coordinates in the first year were set to be (0, 0). (TIFF) [file pcbi.1012893.s002.tiff]

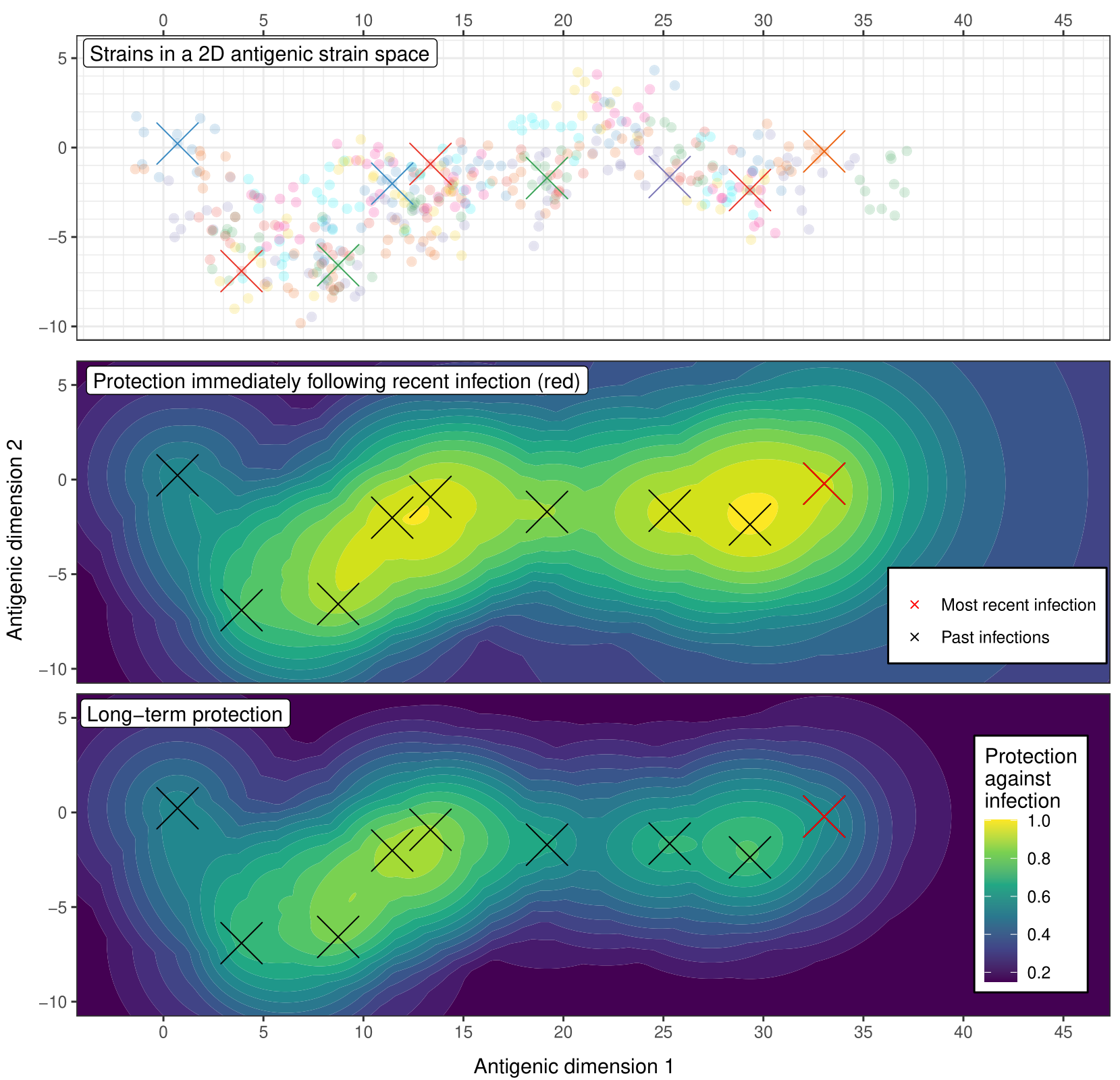

Supplement: S3 Fig — Strains are located on a two-dimensional antigenic map. The antigenic distance between two strains quantifies their dissimilarity, with one unit of antigenic distance corresponding to a two-fold dilution in hemagglutinin inhibition titre. The simulated infections of a single individual are indicated by crosses. The infection furthest along antigenic dimension 1 is the most recent infection. (Middle and bottom panels) The estimated protection against infection from a strain of influenza, as a function of the strain’s location on the antigenic map. The middle panel shows the protection against infection immediately following the most recent infection (red cross), a combination of the long-term immune responses of all past infections (black crosses and red cross), and the short-term immune response of the most recent infection. The bottom panel shows the protection against infection a long time after the most recent infection (any time greater than 1.27 years: the time taken for the short-term immune response to go to 0), a combination of only the long-term immune responses of all past infections. (TIFF) [file pcbi.1012893.s003.tiff]

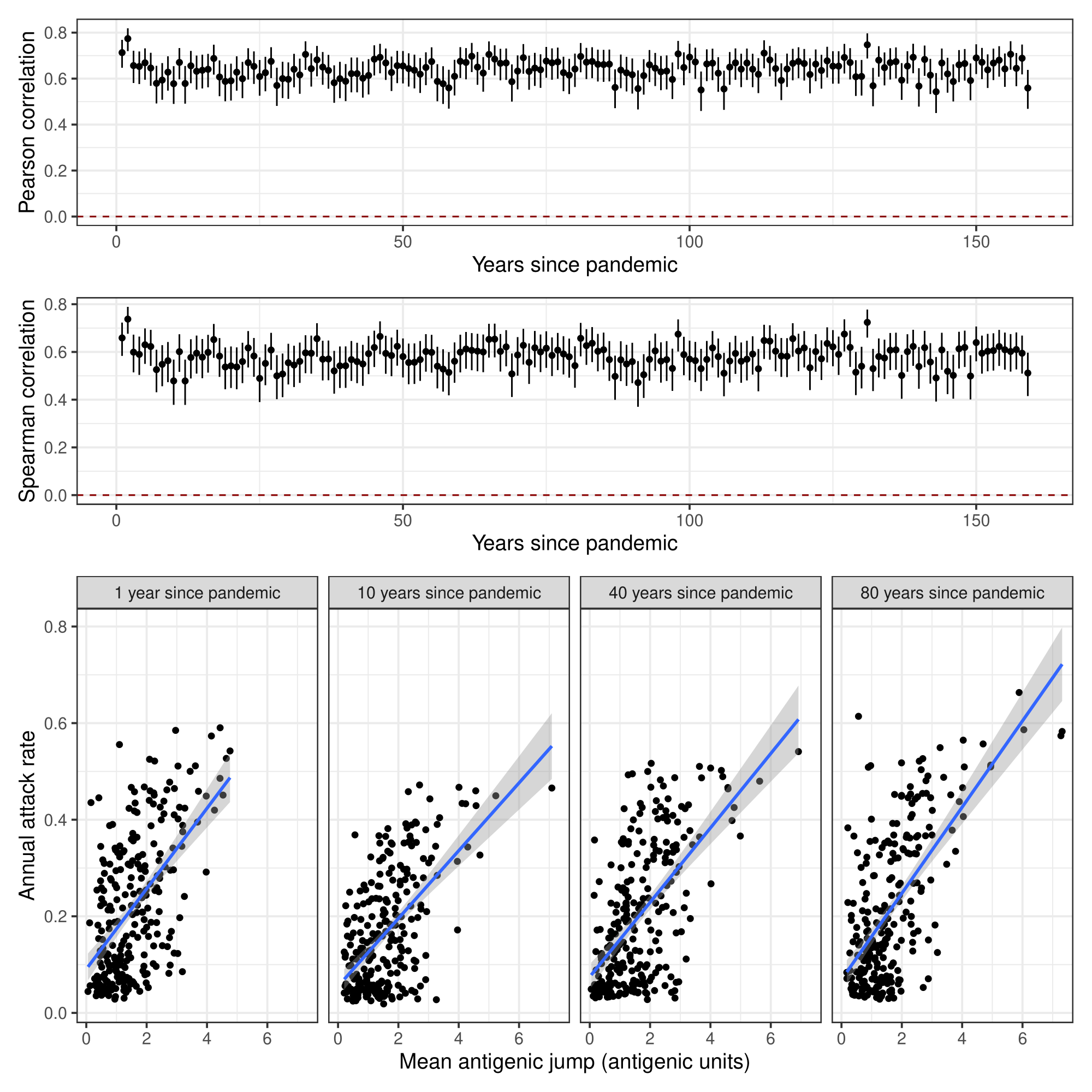

Supplement: S4 Fig — The size of the antigenic jump between years was measured as the distance (in antigenic space) between the mean antigenic coordinates of the adjacent years. (Middle panel) Same as above but for the Spearman correlation. (Bottom panels) The annual attack rate plotted against the size of the antigenic jump for all 256 simulations shown for four different epidemic years. For visualisation of the relationship between the two variables we have also plotted a linear regression line (fitted using ordinary least squares). (TIFF) [file pcbi.1012893.s004.tiff]

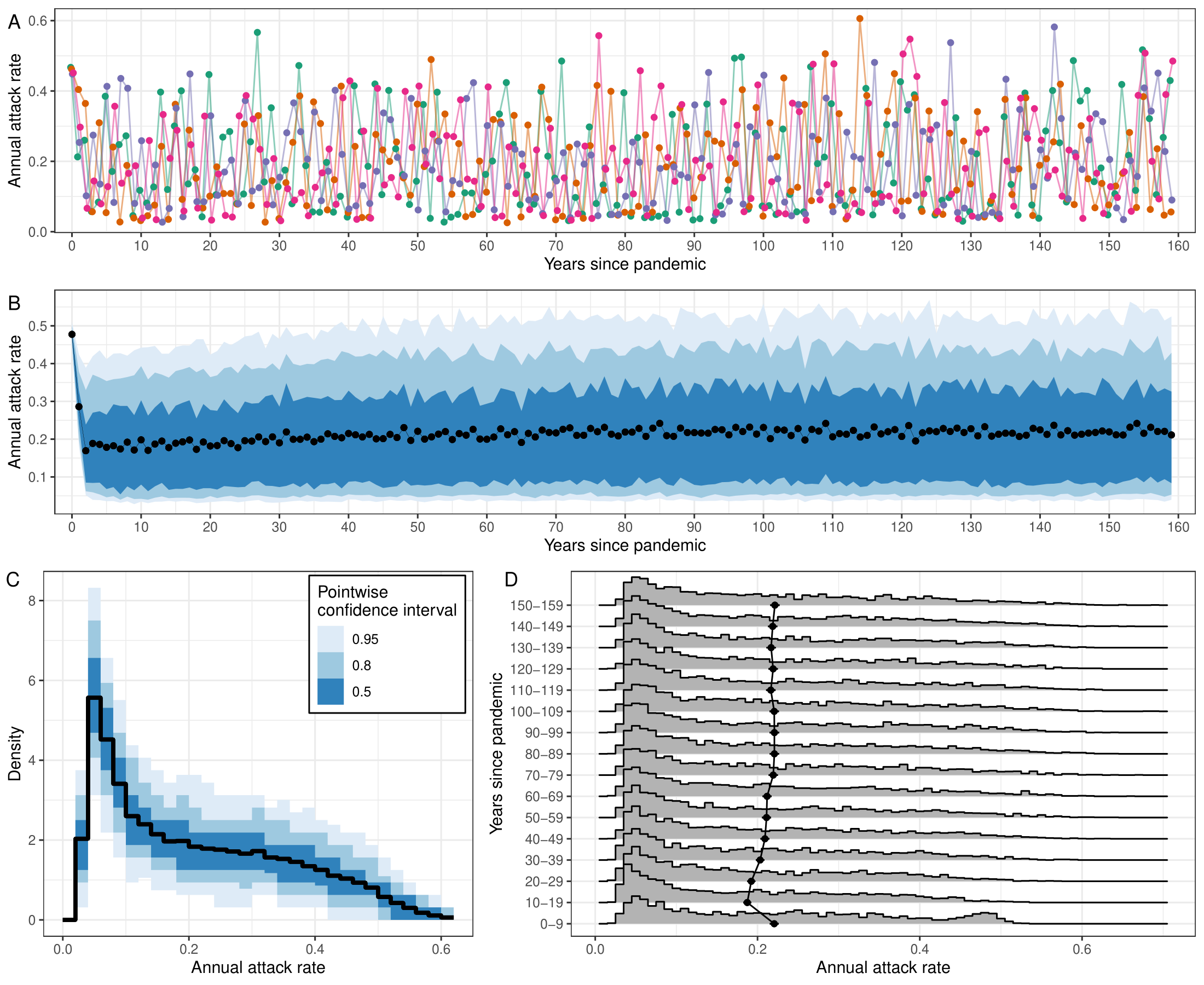

Supplement: S5 Fig — (A) The annual attack rate for each epidemic year (points), including the pandemic year (years since pandemic = 0), shown for four independent simulations (four unique colours and lines). (B) The average (mean) annual attack rate for each epidemic year (points) and the central 50% (dark blue), 80% (blue), and 95% (light blue) tolerance intervals, estimated from 256 simulations. Note that the black line and points are the same as in Fig 3. (C) Probability density of the annual attack rate over all 160 epidemic years of each simulation. The probability density was calculated for all 256 simulations; the median (black line) and 50%, 80%, and 95% pointwise confidence intervals (shaded regions) are shown. (D) Probability distributions (grey shaded regions) of the annual attack rate over all 256 simulations for each decade. Also shown are the average (mean) annual attack rates (points) and their 95% confidence interval (small black bars) for each decade. (TIFF) [file pcbi.1012893.s005.tiff]

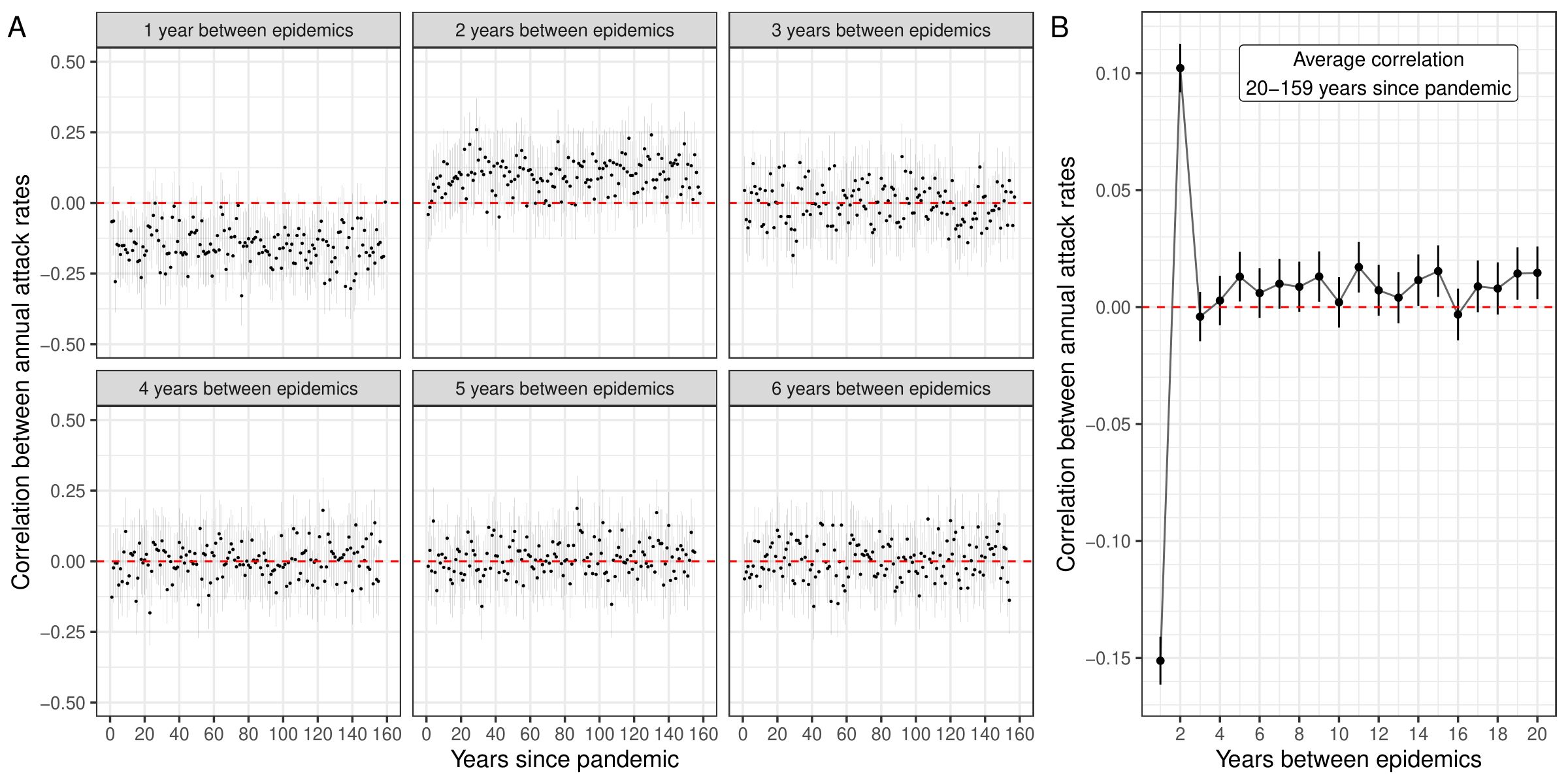

Supplement: S6 Fig — (A) The correlation (points) between the attack rate in a single epidemic year (x-axis) and the attack rate in a future epidemic year (subheadings give time between epidemic years). Each estimate of correlation (points), and its 95% confidence interval (grey lines) is made across 256 simulations. (B) The average correlation of the attack rates between epidemic years (as a function of the number of years between epidemics), estimated over the epidemic years 20–159 years after the pandemic. Estimates are shown with their mean (points) and 95% confidence interval (black bars). The dashed red line in both subplots highlights a correlation of 0 (no correlation). (TIFF) [file pcbi.1012893.s006.tiff]

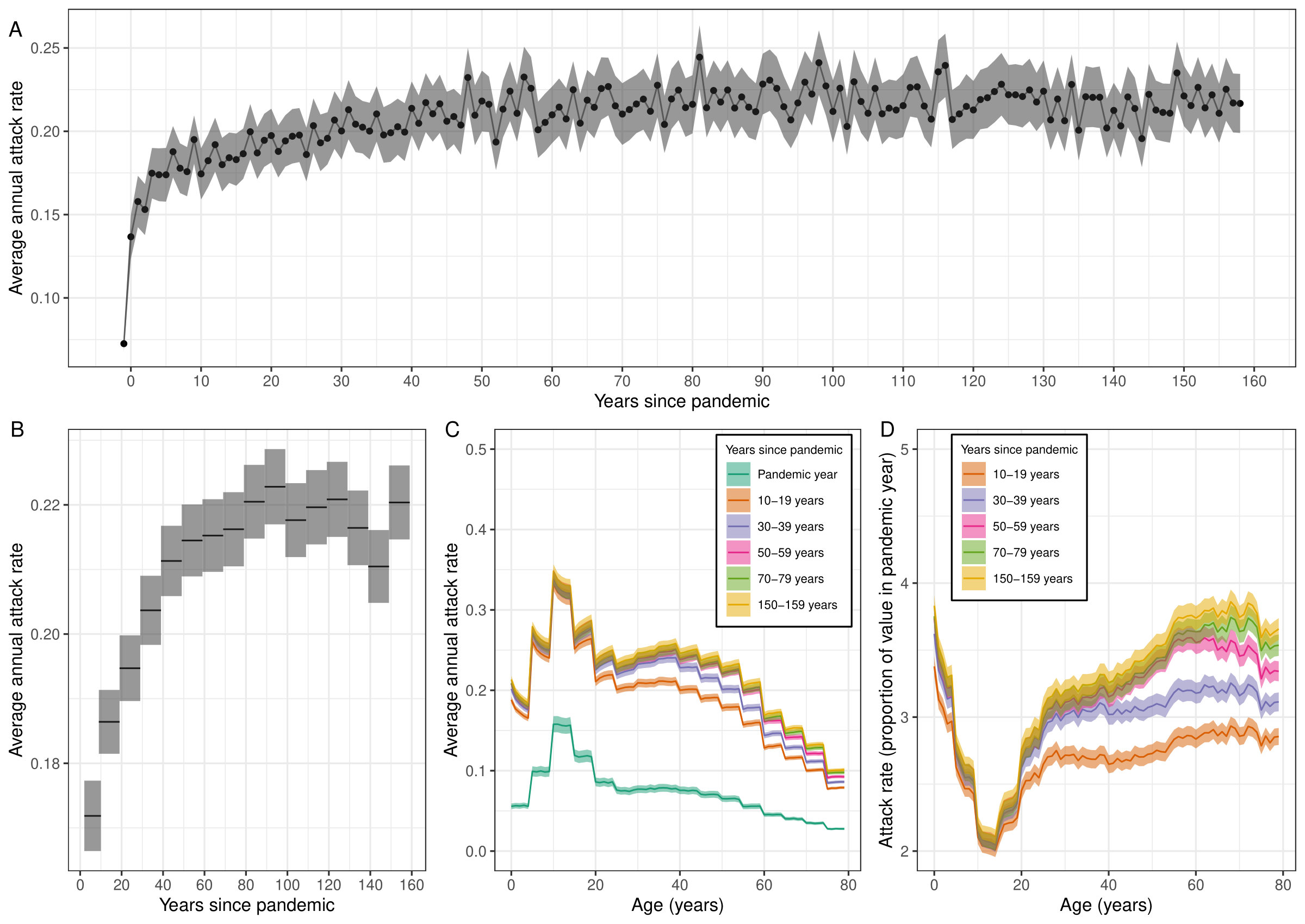

Supplement: S7 Fig — The same as in Fig 3, but simulations were instead run with all individuals having a long-term immune response against the mean antigenic coordinates for the pandemic year (see Methods). (A) Average annual attack rate for each epidemic year, including the pandemic year (years since pandemic = 0). Estimates are shown for the mean (points) annual attack rate and the 95% confidence interval in the mean (shaded region). (B) The mean annual attack rate (black line) and 95% confidence interval in the mean (shaded region) calculated instead for each decade. The first ‘decade’ only includes the years 2–9 years after the pandemic (i.e., does not include the first two years in which the attack rate was still at higher levels). (C) The average annual attack rate by age (0–79 years) for the pandemic year, and for specific decades. Estimates are again shown for the mean (central line) and the 95% confidence interval in the mean (shaded regions). (D) The average annual attack rate by age for the same decades as in (C) but now shown as a proportion of the mean annual attack rate in the pandemic year. (TIFF) [file pcbi.1012893.s007.tiff]

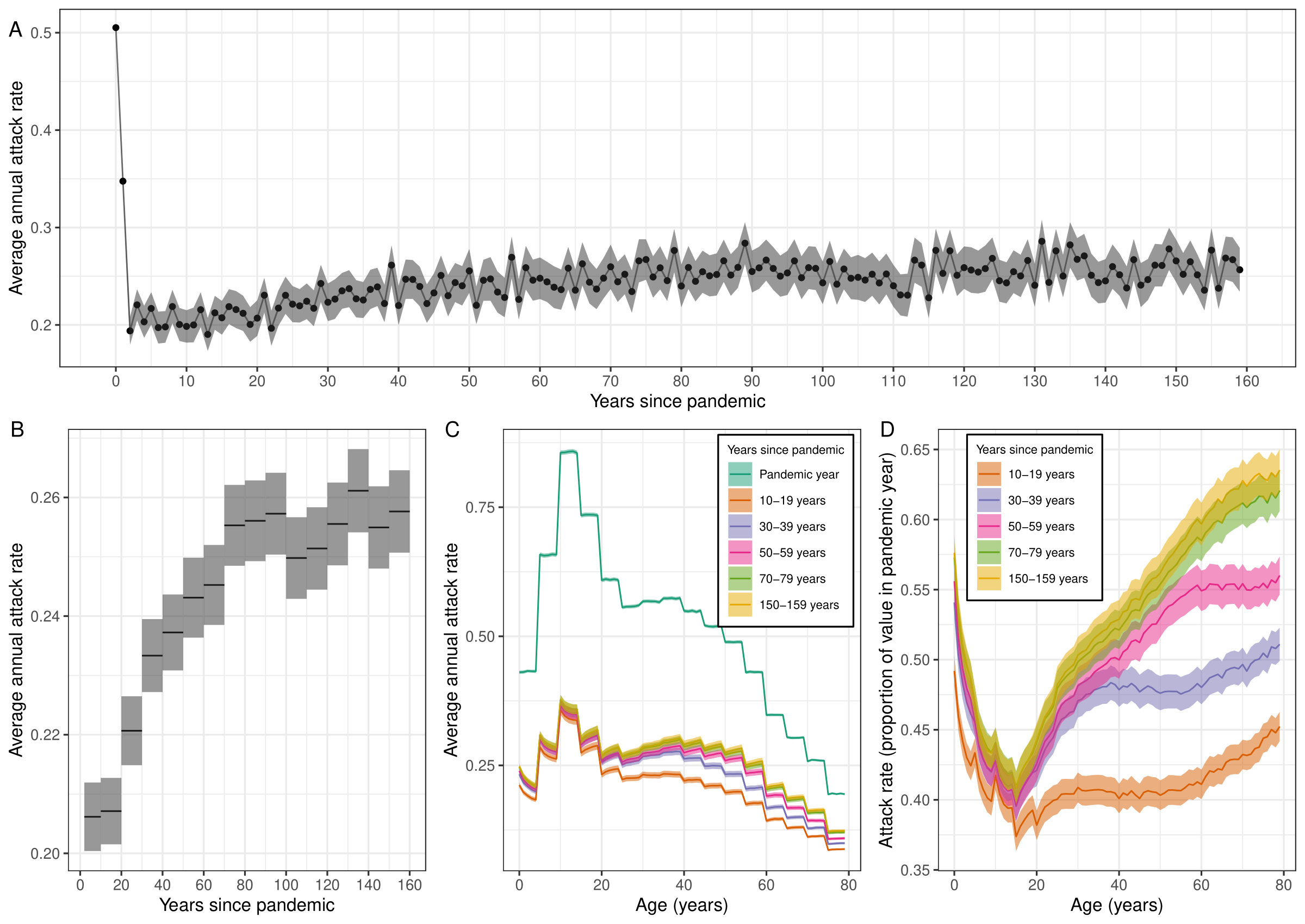

Supplement: S8 Fig — The same as in Fig 3, but simulations were instead run with parameters chosen so the average annual attack rate in the first 40 years was 22.5% (see Methods). (A) Average annual attack rate for each epidemic year, including the pandemic year (years since pandemic = 0). Estimates are shown for the mean (points) annual attack rate and the 95% confidence interval in the mean (shaded region). (B) The mean annual attack rate (black line) and 95% confidence interval in the mean (shaded region) calculated instead for each decade. The first ‘decade’ only includes the years 2–9 years after the pandemic (i.e., does not include the first two years in which the attack rate was still at higher levels). (C) The average annual attack rate by age (0–79 years) for the pandemic year, and for specific decades. Estimates are again shown for the mean (central line) and the 95% confidence interval in the mean (shaded regions). (D) The average annual attack rate by age for the same decades as in (C) but now shown as a proportion of the mean annual attack rate in the pandemic year. (TIFF) [file pcbi.1012893.s008.tiff]

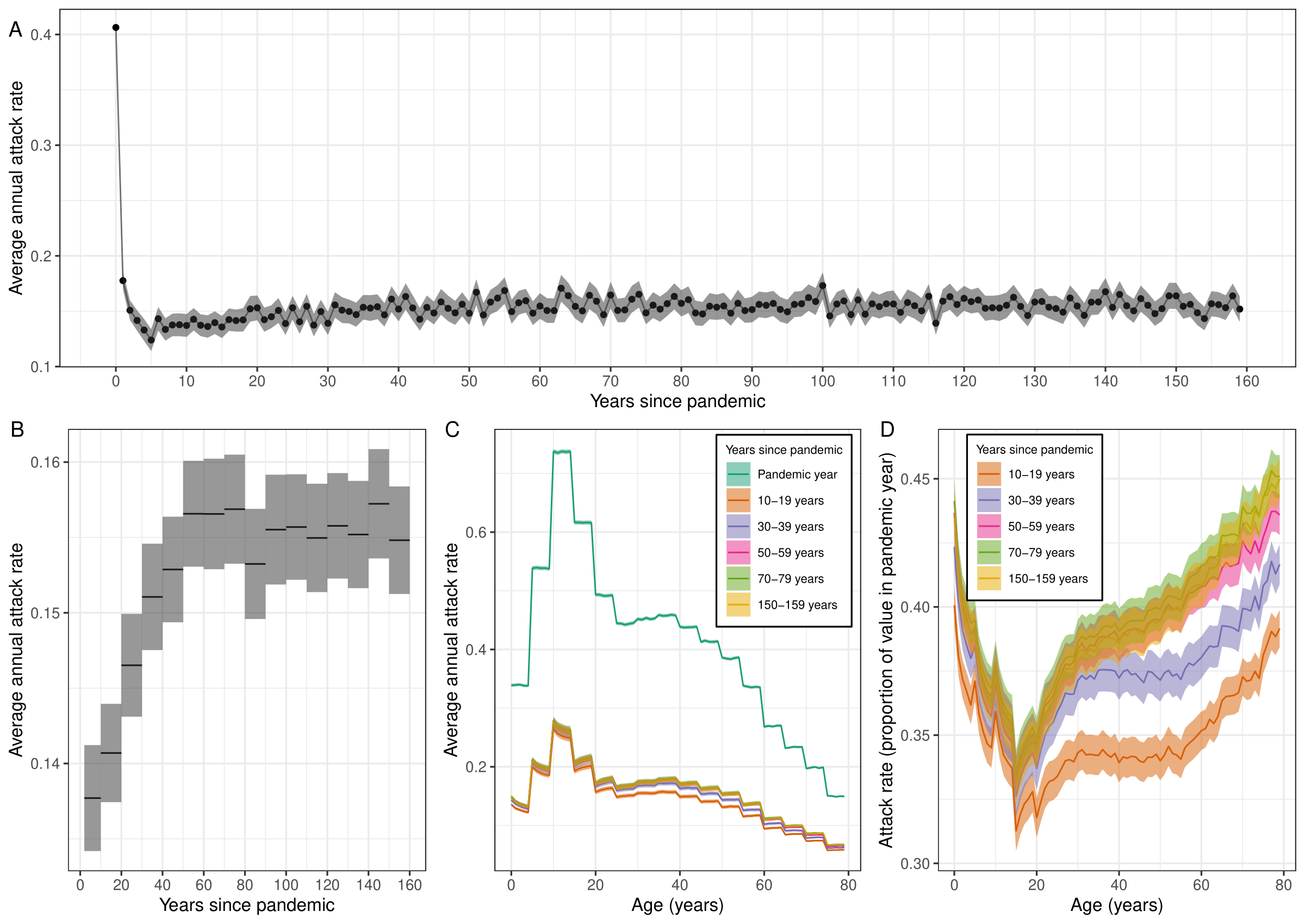

Supplement: S9 Fig — The same as in Fig 3, but simulations were instead run with parameters chosen so the average annual attack rate in the first 40 years was 15.0% (see Methods). (A) Average annual attack rate for each epidemic year, including the pandemic year (years since pandemic = 0). Estimates are shown for the mean (points) annual attack rate and the 95% confidence interval in the mean (shaded region). (B) The mean annual attack rate (black line) and 95% confidence interval in the mean (shaded region) calculated instead for each decade. The first ‘decade’ only includes the years 2–9 years after the pandemic (i.e., does not include the first two years in which the attack rate was still at higher levels). (C) The average annual attack rate by age (0–79 years) for the pandemic year, and for specific decades. Estimates are again shown for the mean (central line) and the 95% confidence interval in the mean (shaded regions). (D) The average annual attack rate by age for the same decades as in (C) but now shown as a proportion of the mean annual attack rate in the pandemic year. (TIFF) [file pcbi.1012893.s009.tiff]

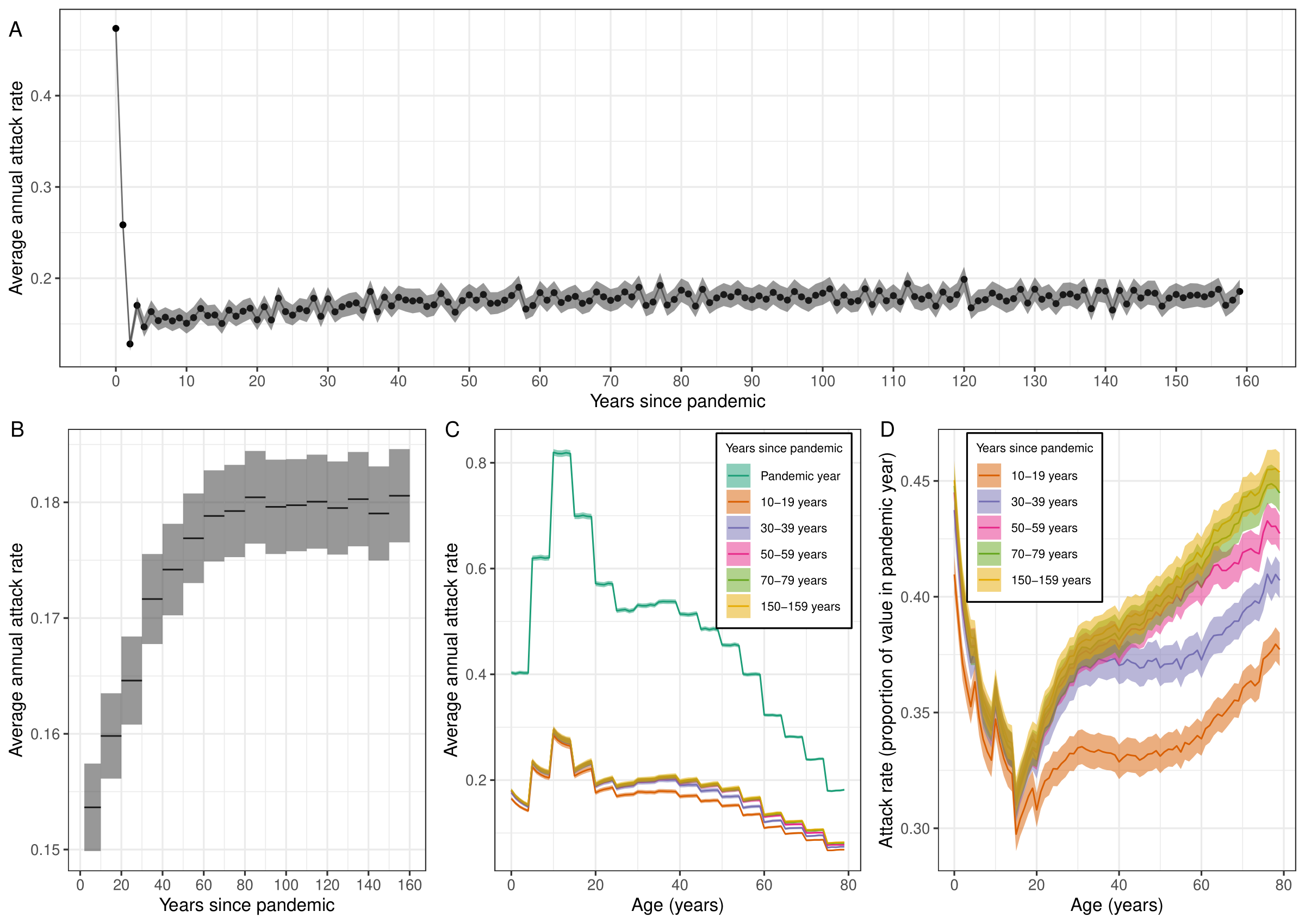

Supplement: S10 Fig — The same as in Fig 3, but simulations were instead run with a global drift model that assumed mean antigenic coordinates drifted along the x-axis at a constant rate with no drift along the y-axis (i.e. no variation in antigenic transitions between years). (A) Average annual attack rate for each epidemic year, including the pandemic year (years since pandemic = 0). Estimates are shown for the mean (points) annual attack rate and the 95% confidence interval in the mean (shaded region). (B) The mean annual attack rate (black line) and 95% confidence interval in the mean (shaded region) calculated instead for each decade. The first ‘decade’ only includes the years 2–9 years after the pandemic (i.e., does not include the first two years in which the attack rate was still at higher levels). (C) The average annual attack rate by age (0–79 years) for the pandemic year, and for specific decades. Estimates are again shown for the mean (central line) and the 95% confidence interval in the mean (shaded regions). (D) The average annual attack rate by age for the same decades as in (C) but now shown as a proportion of the mean annual attack rate in the pandemic year. (TIFF) [file pcbi.1012893.s010.tiff]

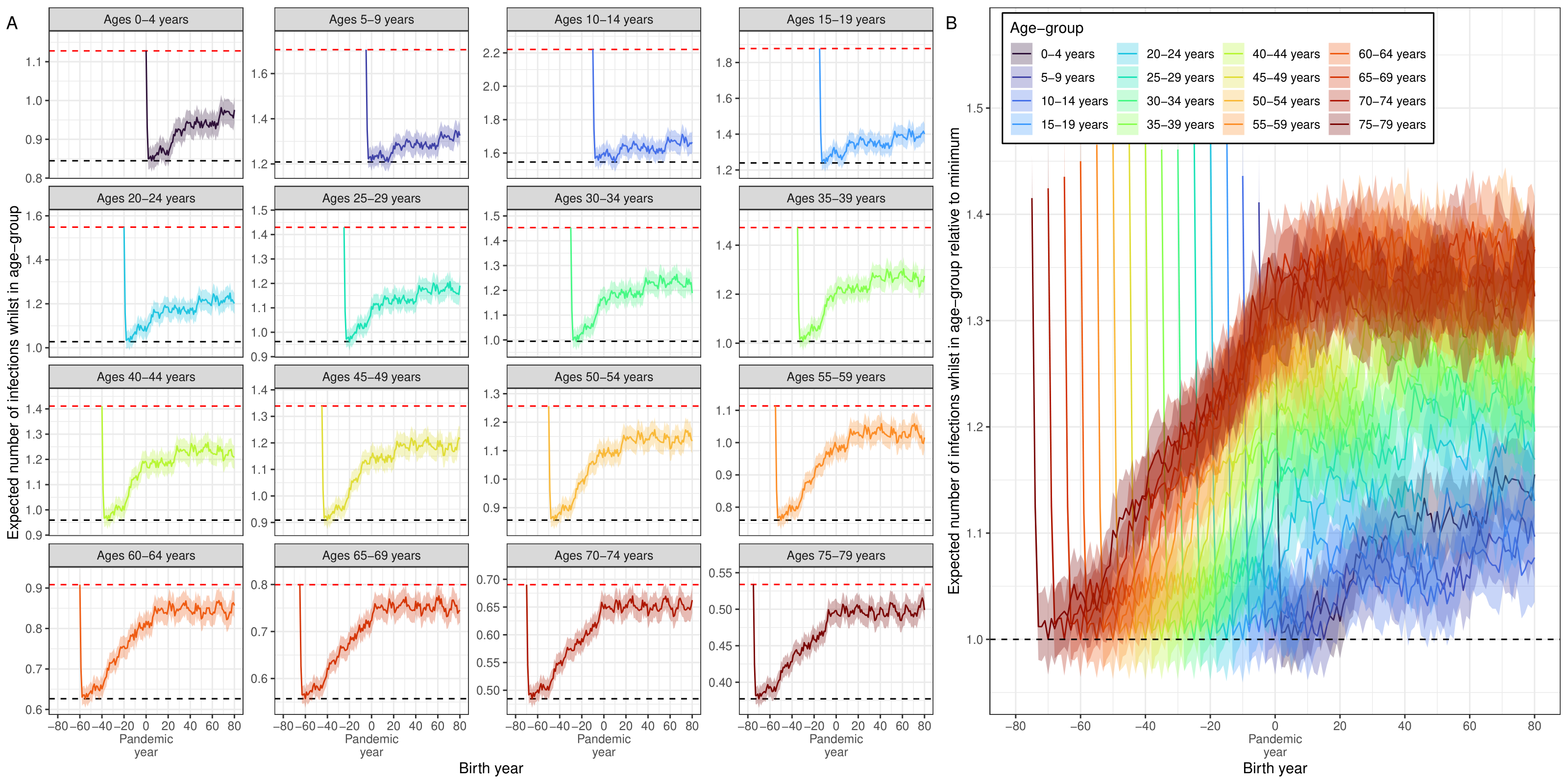

Supplement: S11 Fig — Estimates are only shown for the cohorts in which influenza was circulating all years in which they were in the life-stage. For example, for the life-stage representing infections while aged 20–24 years, only those born 20 years before the pandemic and later are considered. Horizontal dashed lines show the maximum expected number of infections, found for the cohort entering the life-stage in the year of the pandemic (red), and the minimum expected number of infections, found for the cohort entering the life-stage two years after the pandemic (black). (Right) The expected number of infections an individual will experience during different life-stages by cohort relative to the cohorts for which the value (for each life-stage) is a minimum (black dashed line). All estimates are shown with the mean (lines) and the 95% confidence interval in the mean (shaded region). (TIFF) [file pcbi.1012893.s011.tiff]

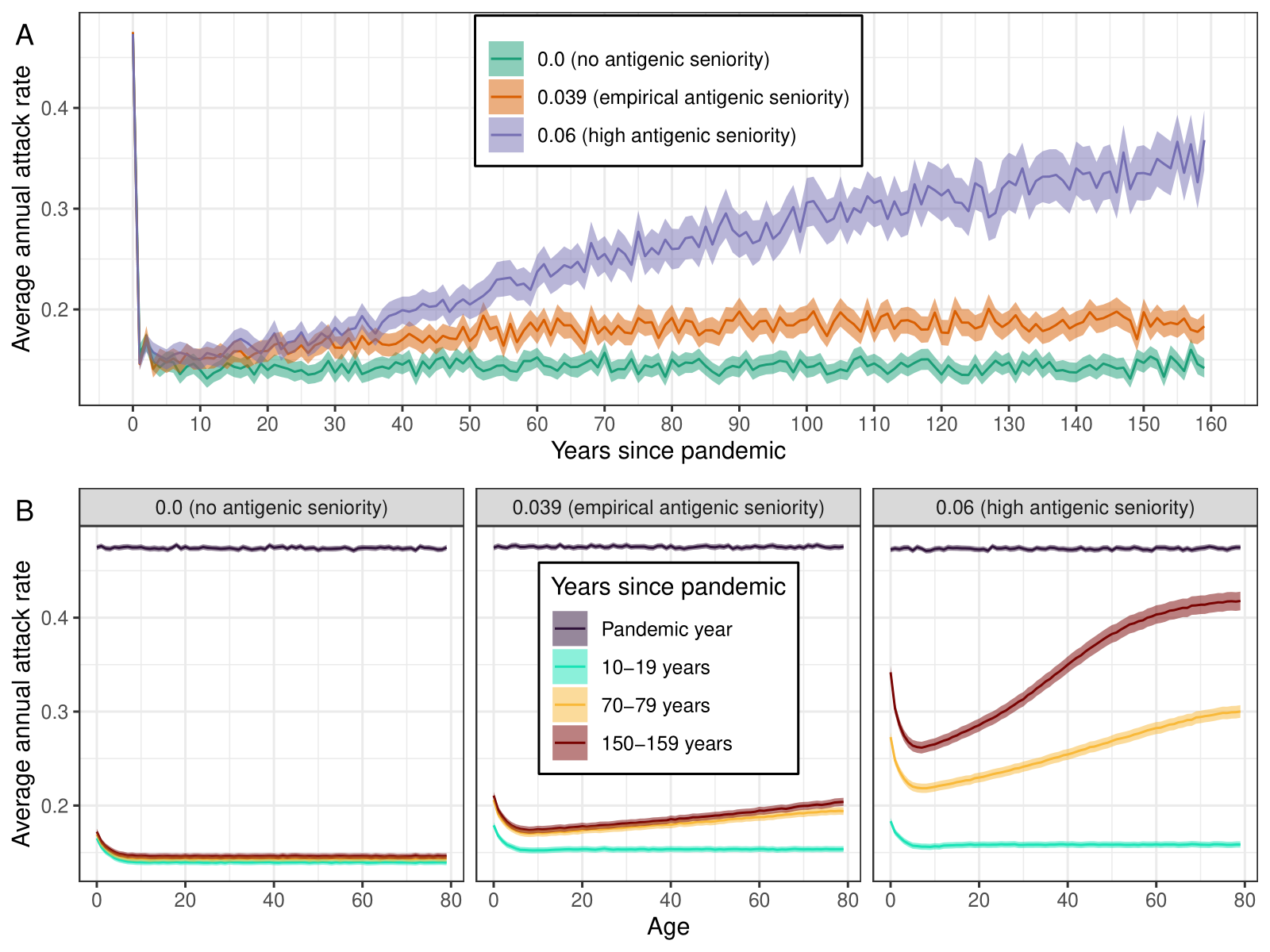

Supplement: S12 Fig — (A) and (B) are the same as in Fig 4, but the age-contact matrix now assumes equal contact rates between all age-groups (the mean contact rate matches the mean contact rate of the age-contact matrix used in the main analysis). (A) The average annual attack rate for each epidemic year, including the pandemic year (years since pandemic = 0), for different values of the antigenic seniority parameter (see Methods). The number of infections until the antibody response is suppressed by 50% is given by 0.5 divided by the antigenic seniority parameter. Estimates are shown with the mean (points) and the 95% confidence interval for the mean (shaded region). (B) The average annual attack rate by age (0–79 years) for the pandemic year, and for specific decades, for different values of the antigenic seniority parameter. Estimates are shown with the mean (central line) and the 95% confidence interval for the mean (shaded regions). (TIFF) [file pcbi.1012893.s012.tiff]

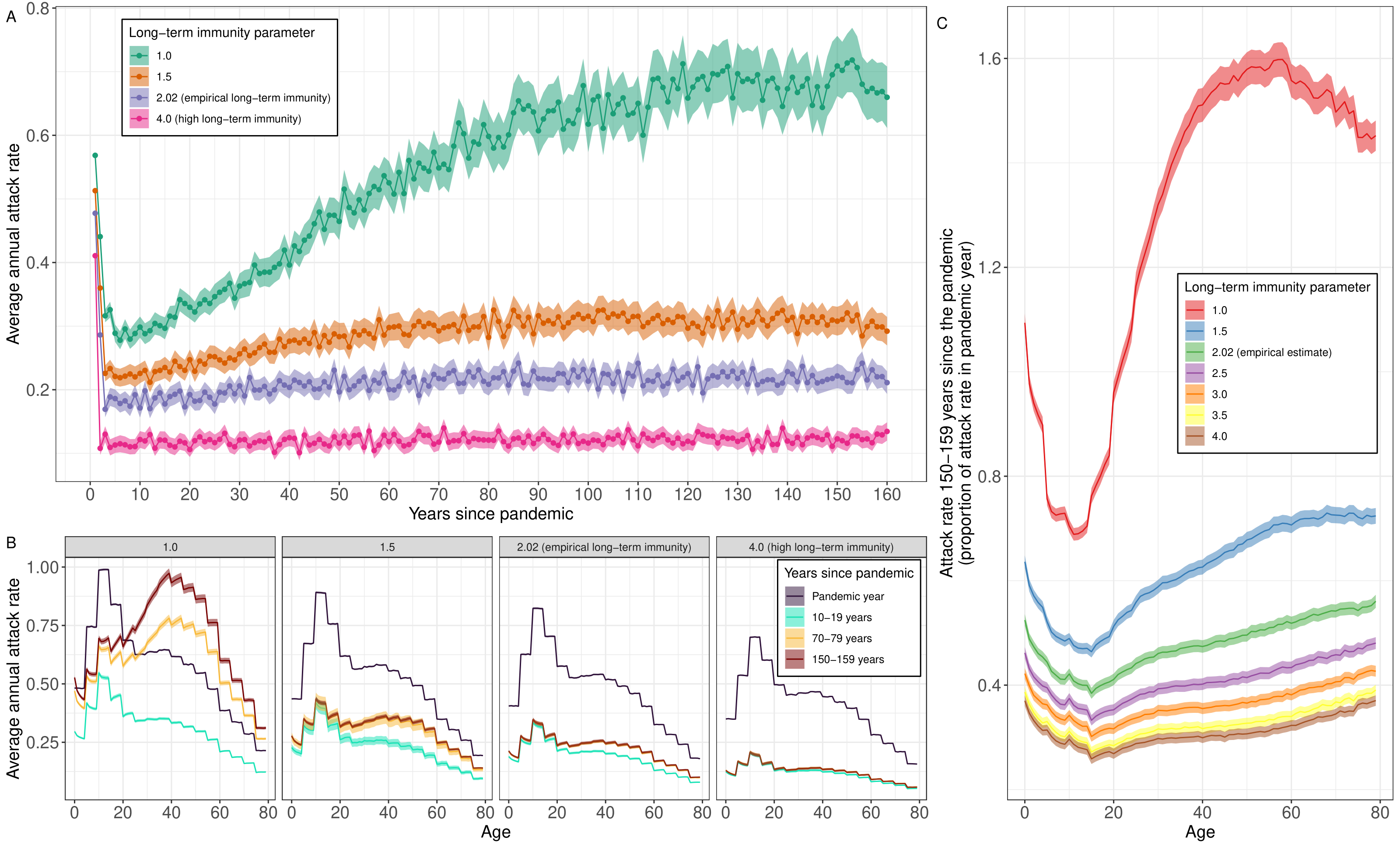

Supplement: S13 Fig — Estimates are shown with the mean (points) and the 95% confidence interval for the mean (shaded region). (B) The average annual attack rate by age (0–79 years) for the pandemic year, and for specific decades, for different values of the long-term immunity parameter. Estimates are shown with the mean (central line) and the 95% confidence interval for the mean (shaded regions). (C) The average annual attack rate by age for the years 150–159 years after the pandemic, shown relative to the average annual attack rate by age in the year of the pandemic, for different values of the long-term immunity parameter. Estimates are again shown with the mean (central line) and the 95% confidence interval for the mean (shaded regions). (TIFF) [file pcbi.1012893.s013.tiff]

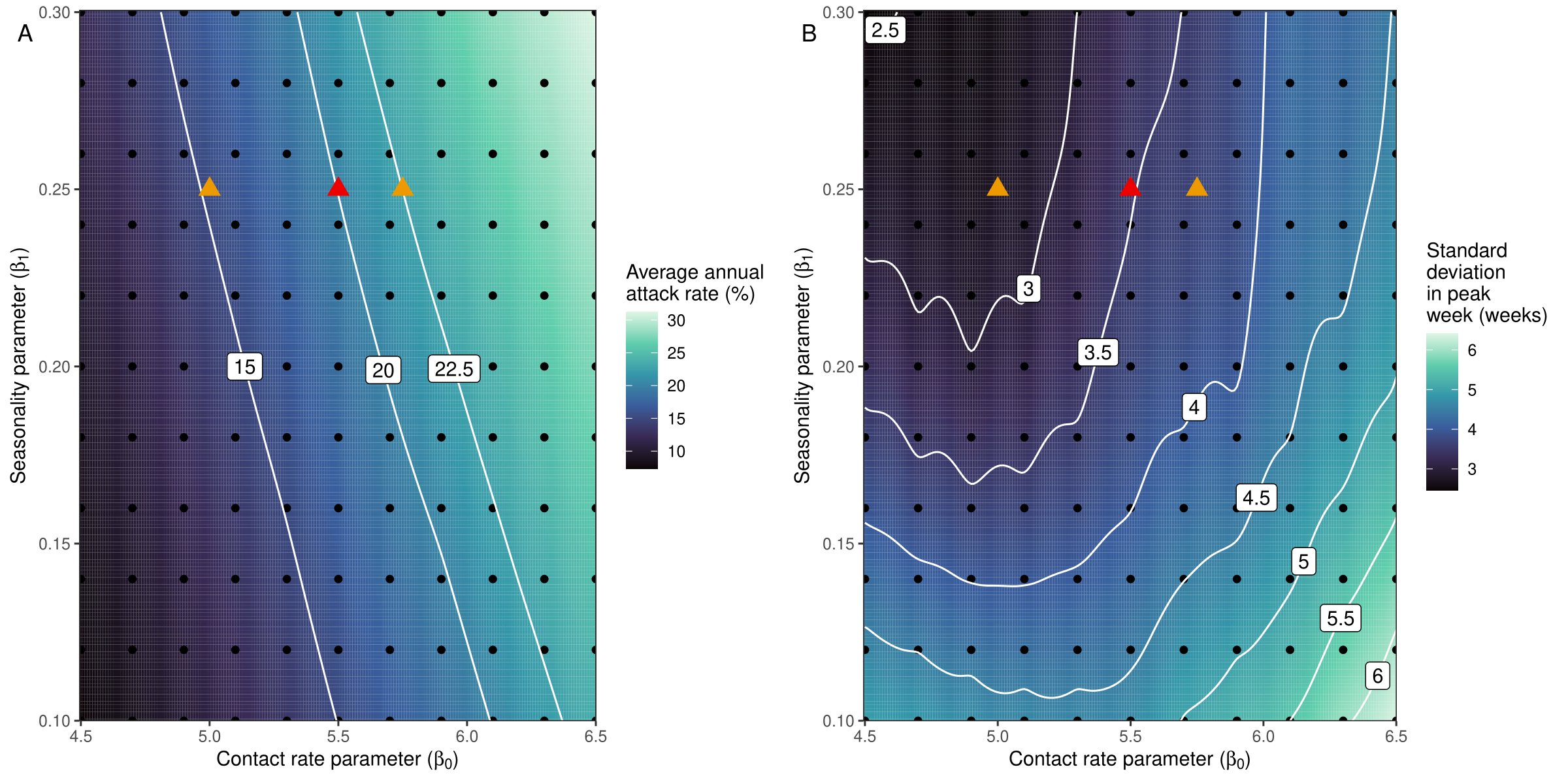

Supplement: S14 Fig — (A) The average annual attack rate over the first 40 epidemic years (including the pandemic year). (B) The standard deviation in the timing of the peak week over the first 40 epidemic years (including the pandemic year). (A,B) Point estimates were made using the epidemiological model (20 simulations) at set values (grid of black dots) of the contact rate parameter, 0, and the seasonality parameter, 1. Continuous estimates were then made using generalised additive models, with the output variable the point estimates, and the parameter values (in a two-dimensional thin plate spline) as the input variables. Contours are shown (white) for specific values of the continuous estimates. The parameters used for the main analysis (red triangle) were selected so that in the first 40 years the average annual attack rate was approximately 20% and the standard deviation in the timing of the peak week was approximately 3.5 weeks. Parameters for sensitivity analysis (orange triangles) were chosen so that in the first 40 years the average annual attack rate was approximately 15% and 22.5%. (TIFF) [file pcbi.1012893.s014.tiff]

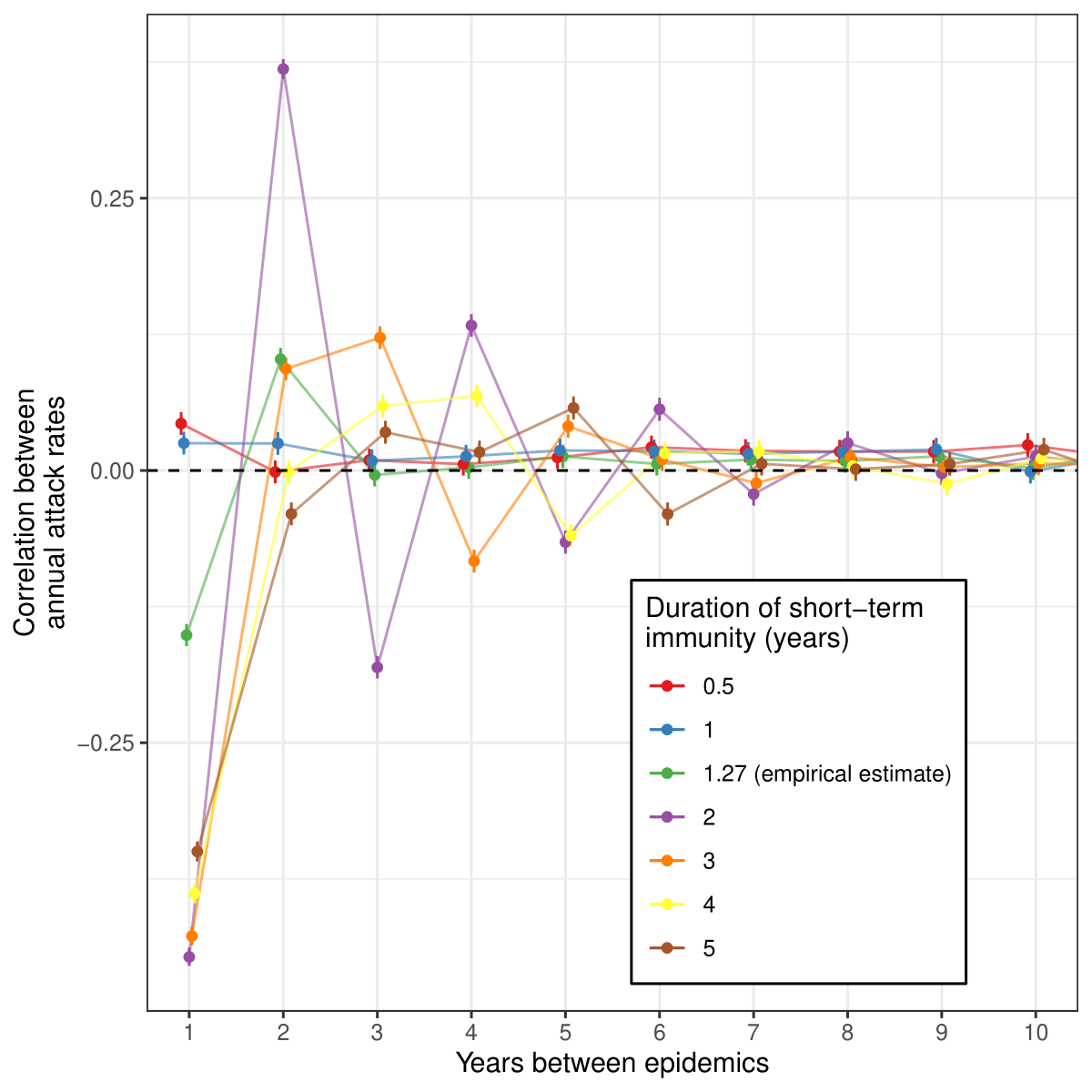

Supplement: S15 Fig — The average Spearman correlation in the annual attack rate between epidemic years (as a function of the number of years between epidemics), estimated over the epidemic years 20–159 years after the pandemic. Estimates are shown with their mean (points) and 95% confidence interval (bars) for different durations of short-term immunity (colours, see legend). The dashed black line highlights a correlation of 0 (no correlation). (TIFF) [file pcbi.1012893.s015.tiff]
